# Cross-country information transmissions and the role of commodity markets: A multichannel Markov switching approach

**DOI:** 10.1371/journal.pone.0202251

**Published:** 2018-08-16

**Authors:** Carl-Henrik Dahlqvist

**Affiliations:** 1 LFin, Louvain School of Management, Université catholique de Louvain, Louvain-la-neuve, Belgium; 2 CeReFiM, University of Namur, Namur, Belgium; 3 NaXys, University of Namur, Namur, Belgium; Central South University, CHINA

## Abstract

This paper examines the interrelationships among 9 advanced economies using a novel multichannel approach to investigate, beyond the usual causal relationships, the time-varying dimension of the channels conveying causal relationships. The model is derived from the well-known Markov Switching setting and account for systems described by multiple variables. A Markov switching causality measure is adapted to account for information transmissions between distinct multivariate systems. Each country is described by 5 different fundamental variables reflecting its state. Our multichannel causality measure is then applied on these sets of time series to determine, over time, the main channels through which the information is transmitted between the different countries. In a second step, we investigate the relationships existing between these countries and the commodity markets and look at the possible use of the commodity markets as an indirect channel of information transmission between countries.

## Introduction

Throughout the last decade, new methodologies and approaches have been developed to better understand the interconnections existing between parts of the global economic system, especially after the last financial crisis. Indeed, the greater interdependence of markets across the globe explained by the financial globalization, and the financial crisis of 2008 highlighted the necessity for a rethinking of economic and financial policies which requires a better understanding of the mechanisms governing information transmission inside the economy. This transmission mechanism was investigated from a number of perspectives, relying mainly on causality analysis, for different markets and asset classes such as the equity market [1–4], the financial market [5, 6], the sovereign bond market [7–9] or the commodities [10–13].

The literature related to interrelations focuses primarily on the detection of stress transmissions often identified as contagion. Contagion may be defined as cross-country transmissions of shocks or more generally as cross-country spillover effects. As recalled by [4], the current literature identifies two possible theories of contagion. The first theory defines the interdependence of economies through real and financial linkages such as flows of goods, service and capital, as the main carrier of information. The second strand of the literature argues that stress transmission occurs from one country to another due to the lack of anticipation of investors having incomplete information. Indeed a shock in one country may trigger a reassessment of the risks in other countries by international investors [14, 15]. This risk reassessment also called “Wake up call” in [16] increases the relative importance of domestic fundamentals in the transmission of stress during period of turmoil.

We propose in this paper to consider a more broad connectedness measure that does not only consider shocks in order to infer the causal relationships that we identify as information transmissions. We rely on a new framework inspired by the concept of causality developed by [17] which has already been extensively used to study the interrelations existing inside the economic system (see [18–20] among others). However, in contrast with the current literature which often considers one type of asset or one market at a time, we propose to consider several types of asset at the same time to estimate more globally the transmission of information between countries. Each country is therefore characterized by a set of financial variables on which a novel multichannel causality test is applied.

In our multichannel framework, a channel is defined as a pair of parameters characterizing the state of two different systems. Each system being described in terms of several parameters, different channels may possibly convey causal relationships over time. In the proposed application, the fundamentals represent the different parameters while the considered systems are countries. The set of selected fundamentals has been therefore chosen so as to proxy the state of each country economy. The objective of the paper is therefore not the identification of contagion periods but rather the analysis of the evolution of the channel through which the information is transmitted depending on the period considered: crisis period vs. non crisis period. We consider thus not only stress transmission but more broadly information transmission, i.e. inter-dependencies affecting either positively or negatively countries, and analyze how the channels through which the information transits evolve in period of turmoil where contagion may be observed.

To empirically assess the relative contribution of several transmission channels over time, our econometric framework should accommodate two important features: (i) multivariate dimension and (ii) a time varying dimension. Inspired by the work of [21, 22] who adapted the univariate Granger causality test to the multivariate case, we propose an extension of the Markov regime switching Granger causality test developed by [23] to account for information transmission between distinct multivariate systems. In our model, each regime corresponds to a specific pair of variables including one of the financial variables describing the country receiving the information, i.e. the dependent variable and one of the financial variables describing the country transmitting the information, i.e. the explanatory variable. The proposed framework enables us to go beyond the usual estimation of cross-country inter-dependencies by considering multiple explanatory and dependent variables describing two different systems. This method allows us to define both the existence of a causal link and the main channels through which the information transit at each time step.

Our empirical investigation starts by the analysis of cross-country information channels. We consider five different measures of economic wealth for each country: an equity index, an index representing the main financial institutions of the country, the sovereign debt returns, the exchange rate of the local currency and a measure of the volatility inside the equity market. These indicators regroup most of the financial variables extensively used in the literature to treat inter-dependencies between countries. Aside from the identification of cross-country connectedness, the aim of the paper is also to consider the interactions existing between countries and the commodity markets and determine if commodities could be considered as an additional indirect channel used to transmit information from one country to another.

Overall, the paper contribution to the literature is mainly threefold. First, we propose a multichannel approach which is able to define the existence of causal relationships between distinct complex systems such as countries which could be characterized by several features (e.g. equity indices, sovereign bonds). This approach provides also a clear view on the dominating channels of transmission over time. Secondly, we propose an empirical application of this model to assess the evolution of the relationships existing between countries forming the world major economies. Different financial variables are considered to identify potentially different channels of information transmission, focusing our attention on the global financial crisis and on the European sovereign debt crisis. Third, we include in our investigation the effect of the commodity markets as it plays a key role in both the real economy and the financial system.

The paper is structured in the following manner. The second section presents the multichannel causality measure deriving for the Markov switching setting. In the third section, we present the data-set used in the empirical study, outline the methodology and present the results for the model including only the countries and the model including the additional effect of the commodity market. We finally concludes on the main contributions of the paper in the last section.

## Methodology: MultiChannel Markov switching Granger causality

The concept of Markov-switching regressions was first proposed in econometrics by [24] and then [25] who introduced the estimation via a likelihood function. The formulation we use in this paper derives directly from the work of [26, 27] who developed an iterative inference algorithm, namely the Markov switching model. This approach is one of the most popular nonlinear time series models in the literature and many variants have been proposed ever since. The model, we develop in this paper, is and extension of a Vector Autoregressive (VAR) variant of Hamilton’s model proposed by [28] and then extended by [23] for causality detection using the approach of [17]. The proposed approach being based on the concept of causality developed by [17], we draw away from the “a priori structural” approach (e.g. instrumental variables) that have been used extensively in the microeconometrics literature (see [29] and [30] for surveys). Indeed Granger’s approach may be qualified as an “inferential process” approach, as it relies directly on the data (“inferential”) and on the asymmetry of causality stressed by the condition of Hume “The cause must be prior to the effect” (“process”) [31] to estimate the causal relationships. However, the interest for Granger based approaches in economics and finance has increased in recent years [6, 18–20].

We consider, in our model, two systems X and Y characterized each by a set of time dependent variables $x_{t}=\left\{x_{1,t},\cdots ,x_{N,t}\right\}\in R^{N\times T}$, with N defining the number of parameters characterizing the system X, and $y_{t}=\left\{y_{1,t},\cdots ,y_{M,t}\right\}\in R^{M\times T}$, with M defining the number of parameters characterizing the system Y. The objective of the framework, we developed hereafter, is the detection of a possible transmission of information from system Y to system X and the definition of the favored channel for each time step. The channels are defined as pairs of time series composed of one time dependent variable of system X and another of system Y. If a causal link may be inferred between both variables, the channel is considered as active in the information transfer process. The channels are, nevertheless, not mutually exclusive as for each time step, a probability of activity is provided for each channels.

We start by defining the relationship between our two systems thanks to a multivariate VAR model taking into account both the effects of the past of ***x***_*t*_ and ***y***_*t*_ on the current value of ***x***_*t*_. We see in Eq 1 that both the dependent and explanatory variable are multivariate.

$$\begin{array}{ccc} x_{t} & = & \alpha +\sum_{l=1}^{L}\beta_{l}x_{t-l}+R_{l,t}\Phi y_{t-l}+\varepsilon_{t} \end{array}$$ (1)

Here **Φ** represents the coefficient characterizing the transfer of information from the system Y to the system X and is defined by:

$$\begin{array}{c} \Phi =(\begin{array}{cccc} \phi^{1,1} & \phi^{1,2} & \cdots & \phi^{1,M}\\ \phi^{2,1} & \phi^{2,2} & \cdots & \phi^{2,M}\\ \vdots & \vdots & \ddots & \vdots \\ \phi^{N,1} & \phi^{N,2} & \cdots & \phi^{N,M} \end{array}) \end{array}$$ (2)

with *ϕ*^*i*,*j*^ the coefficient characterizing the relationship between the *j*^*th*^ parameter of the system *Y* and the *i*^*th*^ parameter of the system *X* with a lag of l.

As for the parameter ***R***_*t*_, it provides the information about the active channel at each time step and is given by:

$$\begin{array}{c} R_{t}=(\begin{array}{cccc} r_{t}^{1,1} & r_{t}^{1,2} & \cdots & r_{t}^{1,M}\\ r_{t}^{2,1} & r_{t}^{2,2} & \cdots & r_{t}^{2,M}\\ \vdots & \vdots & \ddots & \vdots \\ r_{t}^{N,1} & r_{t}^{N,2} & \cdots & r_{t}^{N,M} \end{array}) \end{array}$$ (3)

Here, all $r_{t}^{i,j}$ are latent random variables that reflect the regime of the system for every time step *t*. Each variable $r_{t}^{i,j}$ takes its value in the set of {0, 1} which implies an information transmission from the variable *j* of system Y to the variable *i* of system X for $r_{t}^{i,j}=1$ and no transmission for $r_{t}^{i,j}=0$. Considering the number of parameters for both systems, there exist *N* × *M* possible states which are represented by a new parameter *S*_*t*_:

$$\begin{array}{c} S_{t}=\{\begin{array}{cc} 1 & \;\text{if}\;r_{t}^{1,1}=1\\ 2 & \;\text{if}\;r_{t}^{1,2}=1\\ \cdots & \\ M+1 & \;\text{if}\;r_{t}^{2,1}=1\\ \cdots & \\ N\times M & \;\text{if}\;r_{t}^{N,M}=1 \end{array} \end{array}$$ (4)

Assuming that ***R***_*t*_ is the realization of a two-state Markov chain, we get (*N* × *M*)^2^ possible states of nature and the probability for each state of nature is given by:

$$\begin{array}{c} Pr\left(S_{t}=r|S_{t-1}=q\right)=p_{q,r} \end{array}$$ (5)

with *q*, *r* ∈ {1, ⋯, *N* × *M*}.

In this framework, the probability of a regime switch depends only on the value of the most recent regime which involves a short term memory, but other specifications may be applied to take into account a longer period of the systems past. Following the definition of *S*_*t*_, we create two new vectors representing the selected variables in the systems X and Y in each state of nature *S*_*t*_, $X_{t}^{S_{t}}$ and, $Y_{t}^{S_{t}}$.

We do not observe *S*_*t*_ directly but only its effect on the behavior of ***x***_*t*_ knowing ***x***_*t*−1_ and ***y***_*t*−1_. The probability density governing ***x***_*t*_ can be fully described by determining the set of parameters in Eq 1, i.e. the intercept ***α***, the auto-regressive coefficient ***β***_*l*_, the causal parameters **Φ**_*l*_, the transition probabilities *p*_*q*,*r*_ and the variance of the Gaussian white noise *ε*_*t*_. Based on this probability law, we can infer the probability of observing each state of nature *S*_*t*_ for every time step using an iterative algorithm. We start by rewriting the probability of observing ***x***_*t*_ in the state r in time step t as:

$$\begin{array}{c} \xi_{r,t}=Pr\left(S_{t}=r|\Omega_{t},\theta \right) \end{array}$$ (6)

Here Ω_*t*_ is the set of information available to describe $X_{t}^{r}$, we get therefore Ω_*t*_ = {***X***_*t*−1_, ⋯, ***X***_*t*−*l*_, ***Y***_*t*−*l*_}. As for ***θ***, it regroups all the parameters of the model ***θ*** = {***α***, ***β***_*l*_, **Φ**, *p*_*q*,*r*_, *σ*}. With *N* variables for the system X and *M* parameters for the system Y, we get a total number of (2 × *N* + *N* × *L* + *N* × *M* + (*N* × *M*)^2^ + 1) parameters to be estimated. The sum of the *ξ*_*r*,*t*_ for the *N* × *M* possible regimes equals unity as they represent all the possible outcomes. We then follow the approach first proposed by [26] and apply an iterative procedure to infer the *ξ*_*r*,*t*_ for all the time step t. Each iteration involves the estimation of a likelihood function which represents the weighted probability of observing the different possible outcomes considering the set of estimated parameters and the past realizations of the system. The likelihood function is defined as a conditional density of ***x***_*t*_ in the following manner:

$$\begin{array}{c} f\left(x_{t}|\Omega_{t-1},\theta \right)=\sum_{q}^{N\times M}\sum_{r}^{N\times M}p_{q,r}\eta_{r,t}\xi_{q,t-1} \end{array}$$ (7)

where *η*_*r*,*t*_ gives the Gaussian density of ***x***_*t*_ for the *N* × *M* different regimes:

$$\begin{array}{c} \eta_{r,t}=f\left(x_{t}|S_{t}=r,\Omega_{t-1},\theta \right)=\frac{1}{\sqrt{2\pi }\sigma }exp[-\frac{X_{t}^{r}-\sum_{l=1}^{L}\beta_{l}X_{t-l}^{r}-\phi^{r}Y_{t-l}^{r}-\alpha }{2\sigma^{2}}] \end{array}$$ (8)

with *α* acting as the mean of the probability density.

We are then able to estimate the probability of observing the system in the state r at time t, based on the likelihood function, the transition probabilities *p*_*q*,*r*_, the probability of observing the system in the different possible state q at previous time step t-1, and the Gaussian density of ***x***_*t*_ for the different regimes. As can be seen from Eq 9, the probability *ξ*_*r*,*t*_ represent simply the sum of the probability of observing the final state r considering all the possible initial state q at time t-1, weighted by their likelihood *η*_*r*,*t*_ and then divided by the total weighted probability of observing every possible final states (the likelihood function).

$$\begin{array}{c} \xi_{r,t}=\frac{\sum_{q}^{N\times M}p_{q,r}\eta_{r,t}\xi_{q,t-1}}{f(x_{t}|\Omega_{t-1},\theta )} \end{array}$$ (9)

By performing this iteration from t = 1 to t = T, with T the length of the time series characterizing the two systems, we get the following global log-likelihood function:

$$\begin{array}{c} log[f(x_{1},x_{2},\cdots ,x_{T}|x_{0})]=log[\sum_{t=1}^{T}f\left(x_{t}|\Omega_{t-1},\theta \right)] \end{array}$$ (10)

We then maximize the resulting log-likelihood function to obtain the optimal set of parameters ***θ***, i.e. the one that minimizes the most the error terms of Eq 1 weighted by the probabilities of the states of nature *S*_*t*_ instead of the latent factor *R*_*t*_. The maximization of the resulting log-likelihood function is done via a quasi-Newton optimization algorithm based on the Broyden-Fletcher-Goldfarb-Shano secant to update the Hessian but other optimization algorithm may be considered. The value of *ξ*_*r*,*t*_ is then inferred for each time step t using this set of optimal parameters. The statistical inference of a causal link between the system Y and X is finally performed via the F-stat of the estimated *ϕ* which provides information about the existence of a transmission of information for each considered channel. In contrast with [21], we do not get a global statistic of the existence of a causal link between the two systems but one value per channel which could be considered as more precise. In addition, we are able to define at each time step the probability of a transfer through every possible channels. A multivariate extension of the causal time varying VAR approach of [32] could also be considered as an alternative to this procedure. Nevertheless, our approach is model free and is able to treat more complex transition patterns between regimes compared to the single smooth transition considered in [32]. It provides also, in a single estimation, information about the existence of a causal relationship for every channels and their relative activity in terms of information transmission for each time step.

As can be seen from Eqs 10 and 9, we have to initialize the algorithm by defining *ξ*_*q*,0_ for the step *t* = 1. If we assume that the considered Markov chain is ergodic, we can simply set *ξ*_*q*,0_ = *Pr*(*S*_*t*_ = *q* ∣ Ω_0_, ***θ***) equal to the unconditional probability *ξ*_*q*,0_ = *Pr*(*S*_*t*_ = *q*). Following the approach proposed by [27], theses initial probabilities may be estimated from the (*N* × *M* + 1)th row of the matrix (*A*^*t*^ *A*)^−1^ *A*^*t*^ with *A* defined as:

$$\begin{array}{c} A=(\begin{array}{c} I_{N\times M}-P\\ 1^{\prime } \end{array}) \end{array}$$ (11)

with P the matrix of *p*_*q*,*r*_ and *I*_*N*×*M*_ a diagonal matrix of dimension *N* × *M*.

## Results and discussion

### Cross-country information transmissions

As recalled by the numerous contributions related to risk transmission (see [1, 2, 6–8, 33] to quote only a few) several channels of information transmission exist between countries, such as their stock market, financial institutions, sovereign bond yield, exchange rate or interest rate among others. We have selected, in this paper, five different high frequency, i.e. daily, variables reflecting the overall state of a country: the main stock market index, an index regrouping the main financial institutions of the country, a volatility index, the exchange rate and the 5 years USD denominated sovereign bond yield. Most of the selected financial variables have already been extensively used in the literature to treat interdependencies between countries.

Related studies are [1] or [33] which find empirical evidences of stress transmission between international equity markets or [3] which finds evidence of contagion for most of the developed and emerging countries during the 2008 financial crisis. They demonstrate the ability of stock markets to reflect the information transmission between countries. This ability to represent cross-country relationships comes primarily from the fact that stock markets reflect the evolution of a country production capacity both in terms of goods and services and could therefore assess to some extend the evolution of the country balance of payment as the exchange rate does also. With regards the exchange rate, we also included it in the set of parameters for its close link with the commodity markets and it will prove to be an important driver of the interdependencies between countries and commodity markets.

System wide dependencies have also been analyzed for financial institutions. Hence, a growing body of the current literature on contagion and network theory tries to understand the main characteristics of the evolution of the connectivity inside the financial market during the crisis of 2008 with contribution such as [5, 6, 34, 35]. Indeed, the financial crisis highlighted the propensity of financial institutions risk to spill over to their environment and across border. This high level of interdependency may be explained partly by the contractual linkages existing between this type of firms which increase the counterparty risk. In addition to these spillover effects, the interplay between fiscally strained sovereigns and stressed banks worsen the condition of the economy. Indeed, strained public finances have limited the ability of some countries to support their financial system through bailout. Fragile banking systems provided then less support to the economic activity, which in turn further strains public finances. These inter-dependencies between banks and sovereigns demonstrate the importance of considering the impact of external factors on a country’s banking system. We consider therefore the evolution of the banking system as a relevant variable to be considered in our multichannel analysis.

The European sovereign debt crisis of 2011 is another good illustration of contagion between countries as demonstrated by [7] which conclude that sovereign debt returns are even more correlated than equity returns. This period has been the subject of many studies about risk transmission between European sovereigns ([36–38] to quote a few) and could therefore be a privileged channel during this period of time.

Eventually, the volatility index has been chosen to proxy the risk/stress level of a country in order to assess possible stress spillovers between countries. Overall, the selected financial variables have already proven their ability to describe interdependencies between countries. Nevertheless, the proposed multi-channel approach will challenge this ability by considering together all parameters and provide additional information about the evolution of these parameters relevance.

### Data

In order to have an overview of the information transmission inside the global economy, we have selected 9 of the most important economies in the world with the United States and Canada for the American continent, France, the United Kingdom, Italy and Germany for Europe and China, Australia and Japan for the Asia-Pacific region. All the financial variables are expressed in terms of daily variations. Table 1 reports the stock market and the volatility indices selected for the empirical application but also the financial institutions used to estimate the financial indices when no aggregate indices were available. In this later case, the reconstructed financial indices were computed as the weighted average of the daily returns of the country’s main financial institutions using the market capitalizations as weights. The volatility indices are based on the implied volatility of the selected stock market indices. As for the exchange rate, having included the U.S. in our analysis, we express all currencies in terms of the IMF Special drawing rights (SDR) instead of the usual USD.

**Table 1:** This table reports the list of the stock indices, volatility indices selected for the different countries and the list of banks used for the estimation of the financial indices.

	Stock Index	Volatility index	Financial institutions
U.S.	SP500	VIX	S&P500 Bank
Canada	S&P/TSX60	VIXC	Royal Bank of Canada
			Toronto-Dominion Bank
			Bank of Nova Scotia
			Bank of Montreal
U.K.	FTSE100	VFTSE	HSBC
			Standard Chatered
			Lloyd
			Barclays
			Royal Bank of Scotland
Germany	DAX	VDAX	Commerzbank
			Deutsche Bank
France	CAC40	VCAC	BNP Paribas
			Société générale
			Credit Agricole
Italy	MIB30	IVMIB30	Intesa SanPaolo
			Unicredit
			Generali bank
China	SSE50	VXFXI	Bank of China
			China Construction bank
			Industrial and commercial bank of China
			Agricultural Bank of China
Japan	Nikkei	VXJ	Nikkei Bank
Australia	S&P/ASX 200	AS51VIX	S&P/ASX 200 Bank

Regarding the data-set used to represent the commodity markets, we have selected 8 commodities covering 4 major sectors, precious metals with gold and silver, metal with aluminum and copper, energy with crude oil and food with soybean, wheat and corn. All the data-sets have been taken from Bloomberg and range from January 2001 to June 2016 representing 4030 daily observations, including the end of the early 2000s recession due to the dot-com bubble, the Global Financial Crisis of 2008 and the European Sovereign Crisis of 2011.

### Analysis of cross-country information transmissions

We now turn to the first step of our empirical investigation by looking at cross-country information transmissions. Each financial variables presented in the previous section represents a possible transmitter or receiver of information which implies 25 possible channels between two countries. In order to reduce the dimension of the model to be estimated, we start the estimation by applying a filter which eliminates the most distinct non-causal relationships. This filter consists in a simple Granger causality test which is applied for each pair of countries on the 25 possible channels. The significance level of the causality test is set to 0.01 and if no connections are observed we increase this level to 0.05. The Granger Causality test is applied on the complete time series. Once the active channels have been determined, we apply on it our multichannel causality measure which provides for each channel three different information. The first one is the p-value which gives information about the existence of a causal link for a particular channel. Then it provides, for each time step, **Ξ**_*t*_ which includes the matrix of *ξ*_*q*,*t*_ for every possible channels, i.e. the probability of channels’ activity for each time step. Eventually it gives the *p*_*r*,*q*_ matrix representing the probability of all possible regime switches.

**Fig 1:**
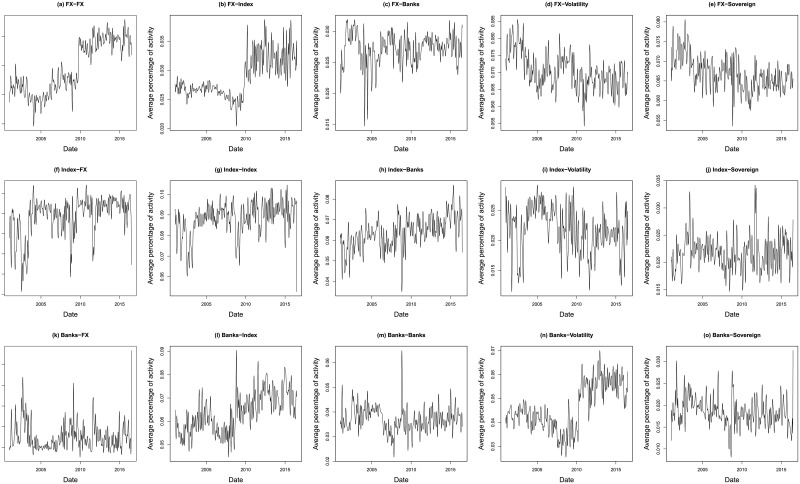
Time evolution of channels activity. These figures report the evolution of the probability of activity for every considered channel. The relative activity has been averaged over the 9 countries of our data-set and then normalized.

**Fig 2:**
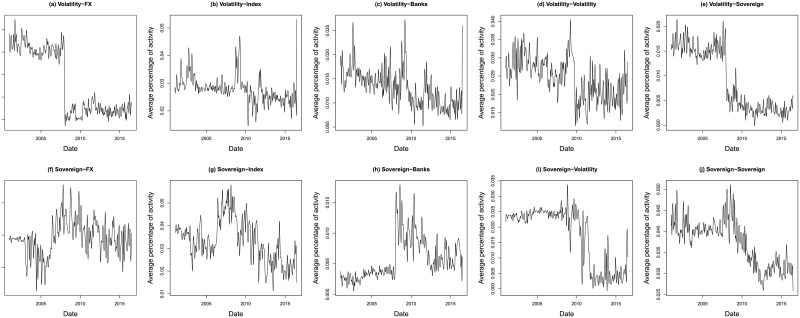
Time evolution of channels activity. These figures report the evolution of the probability of activity for every considered channel. The relative activity has been averaged over the 9 countries of our data-set and then normalized.

Once all these information have been computed for each pair of countries, we first look at the evolution of the global connectivity by regrouping the obtained results for the 9 countries. Figs 1 and 2 report the evolution of the probability of activity for the 25 channels that are considered in this analysis. The activity for each channel has been averaged over the 9 countries and then normalized to properly compare the results. As can be seen from these figures, the probability of channels’ activity seems relatively stable in time. However, we observe for several channels a regime switch occurring mainly during the last financial crisis. The relative importance of the stock markets and financial institutions as transmitter stay stable for the entire data-set while the sovereign bond yield and the currencies become more influential. Surprisingly the volatility indices seem to lose their significance during the crisis period of 2008-2010. Those results highlight the interest of the proposed multi-channel approach which is able to provide new insights regarding the information transmission inside the world economy.

Our analysis relying on a large number of channels, we proposed to aggregate the previous results by considering the four main periods of the recent economic history. We therefore divide the data-set into four periods: the pre-crisis period which goes until the start of the Global Financial Crisis on the 9th of August 2007, the Global Financial Crisis period which goes until the 15th of January 2010, the European Sovereign Debt Crisis which goes until the 31st of October 2013 and finally the post crisis period. This will ease the analysis while still allowing us to look at possible changes in the channels dynamics during these different episodes. To get a more comprehensive and global view, we have again summed and normalized the matrices of probability **Ξ**_*t*_ for the 9 countries and averaged them for the considered periods of time.

The results are reported in Table 2 and show that overall the main channel of cross-country communication seems to be the currencies, with on average 18 percent of the information traveling through this channel. The transfer of information between stock markets represents the second largest channel with around 9 percent overall. The other main channels are the Volatility to Currency, Sovereign bond yield to Currency and Currency to Stock market, Financial institutions to Stock market and Stock market to Financial institutions with on average 6 percent. We see, moreover, that in contrast with the current literature which focuses its attention mainly on the importance of financial institutions on stress transmission, the main carriers of information are rather the currency and the stock market which are both the main receiver and transmitter of information.

**Table 2:** This table reports, for 5 different periods of the recent economic history, the probability of activity of every channels of information considered in this application, averaged on all countries. The vertical axis shows the variables of the country receiving the information and the horizontal axis, the variables of the other country from which the information has been transmitting.

Entire Period					
Global	FX	Index	Banks	Volatility	Sovereign
FX	0,18	0,03	0,03	0,06	0,07
Index	0,06	0,09	0,06	0,02	0,02
Banks	0,02	0,06	0,03	0,05	0,02
Volatility	0,01	0,03	0,01	0,03	0,01
Sovereign	0,01	0,03	0,01	0,02	0,03
Pre-Crisis Period					
Pre-C	FX	Index	Banks	Volatility	Sovereign
FX	0,16	0,03	0,03	0,06	0,07
Index	0,06	0,09	0,05	0,02	0,02
Banks	0,01	0,06	0,03	0,04	0,02
Volatility	0,02	0,04	0,02	0,04	0,02
Sovereign	0,01	0,03	-	0,02	0,04
Global Financial Crisis Period					
Fin-C	FX	Index	Banks	Volatility	Sovereign
FX	0,18	0,03	0,04	0,06	0,07
Index	0,06	0,09	0,06	0,02	0,02
Banks	0,02	0,06	0,03	0,03	0,02
Volatility	-	0,04	0,01	0,03	0,01
Sovereign	0,01	0,04	0,01	0,02	0,04
European Sovereign Debt Crisis Period					
Sov-C	FX	Index	Banks	Volatility	Sovereign
FX	0,19	0,04	0,03	0,06	0,07
Index	0,07	0,10	0,06	0,02	0,02
Banks	0,02	0,07	0,03	0,05	0,02
Volatility	-	0,03	0,01	0,02	-
Sovereign	0,01	0,03	0,01	0,01	0,03
Post-Crisis Period					
Post-C	FX	Index	Banks	Volatility	Sovereign
FX	0,19	0,03	0,03	0,06	0,07
Index	0,07	0,10	0,06	0,02	0,02
Banks	0,02	0,07	0,04	0,05	0,02
Volatility	-	0,03	0,01	0,02	-
Sovereign	0,01	0,02	0,01	-	0,03

As for the evolution in time of the channels’ weight, the results confirms the observation made in Figs 1 and 2 with an increase of the importance of the currencies and the stock markets, both as transmitters and receivers of information, as from the beginning of the financial crisis. For the other channels, we do not see a clear evolution of the repartition, apart from the volatility, which tends to prove that these channels are relatively stable in time.

After having documented the global repartition of the channels and its evolution in time, we may now look in more details at the repartition for each country of our data-set. We use again the information provided by our multichannel causality measure to compute, for each country, the average probability of activity of every channels in a 5 by 5 matrix by averaging the **Ξ**_*t*_ for the whole period. We then consider each country from two perspectives, first as a receiver of information and then as a transmitter. We select, in both cases, the channels for which the observed p-value is under 0.01. Once normalized the resulting two matrices provide, for every countries in our data-set, an overview of the channels used for both information outflows and inflows.

Table 3 provides the resulting channel’s activity index per country, both in terms of inflows and outflows of information. The left part of the table gives the channels through which the information reaches the considered country, with the vertical axis giving the variable of this country, and the horizontal axis, the variable of the aggregated transmitting countries. On the right side of the table, we get the reverse information with the channels through which the information leaves the country, i.e. the variables that impact the most the other countries. The horizontal axis provides now the variables of the country of interest and the vertical axis, the variables of the aggregated receiving countries. As can be seen from theses different tables, the high percentage for the channel Currency-Currency comes mainly from the U.S., Canada, Italy and China for the inflows and from Germany, France, Italy and Japan for the outflows. In contrast with the common idea that the USD is the currency influencing the most the global economy, the results shows on the contrary that the EUR and the JPY lead the information transmission process to the other currencies such as the USD, the GBP and the CNY.

**Table 3:** This table reports for the entire period, on the left, the main channels used for incoming information with the vertical axis reporting the variables of the country receiving the information and the horizontal axis reporting the variables of the other countries from which the information has been transmitted. On the right, we have the main channels used for outgoing information with the horizontal axis reporting the variables of the country transmitting the information and the vertical axis reporting the variables of the other countries to which the information has been transmitted.

U.S.											
IN	FX	Index	Banks	Volatility	Sovereign	OUT	FX	Index	Banks	Volatility	Sovereign
FX	0,48	-	-	0,32	0,11	FX	0,05	0,01	0,10	-	0,11
Index	-	-	-	-	0,02	Index	-	0,02	0,12	0,16	0,12
Banks	-	-	0,02	-	0,05	Banks	-	0,02	0,03	0,02	0,10
Volatility	-	-	-	-	-	Volatility	-	0,01	-	0,03	-
Sovereign	-	-	-	-	-	Sovereign	-	0,08	-	0,02	-
Canada											
IN	FX	Index	Banks	Volatility	Sovereign	OUT	FX	Index	Banks	Volatility	Sovereign
FX	0,48	0,07	-	-	0,24	FX	-	-	-	-	0,14
Index	0,08	-	-	-	-	Index	0,20	0,25	-	0,01	0,08
Banks	-	0,02	-	-	-	Banks	0,14	0,01	0,03	0,09	0,02
Volatility	-	-	-	0,10	-	Volatility	-	0,01	0,01	0,01	-
Sovereign	-	-	-	-	-	Sovereign	-	-	-	-	-
U.K.											
IN	FX	Index	Banks	Volatility	Sovereign	OUT	FX	Index	Banks	Volatility	Sovereign
FX	0,17	0,20	-	0,14	0,01	FX	0,06	-	-	-	-
Index	0,03	0,17	-	-	-	Index	0,04	-	0,14	-	-
Banks	-	0,14	0,06	-	-	Banks	-	-	0,20	0,08	-
Volatility	-	0,01	0,01	-	-	Volatility	0,09	-	-	0,03	-
Sovereign	-	-	-	-	0,06	Sovereign	-	0,13	0,05	0,18	-
Germany											
IN	FX	Index	Banks	Volatility	Sovereign	OUT	FX	Index	Banks	Volatility	Sovereign
FX	-	-	0,06	-	0,27	FX	0,28	0,04	-	-	-
Index	0,14	0,13	-	0,01	0,02	Index	-	0,20	0,10	-	-
Banks	0,03	-	-	-	-	Banks	-	0,07	-	0,08	-
Volatility	-	0,02	0,02	0,10	-	Volatility	-	0,15	0,04	-	0,02
Sovereign	-	0,09	-	0,12	-	Sovereign	-	0,02	0,01	-	-
France											
IN	FX	Index	Banks	Volatility	Sovereign	OUT	FX	Index	Banks	Volatility	Sovereign
FX	-	-	-	-	-	FX	0,40	-	-	-	-
Index	0,22	0,26	-	0,14	0,04	Index	-	0,17	0,07	0,02	-
Banks	0,02	0,09	0,07	0,04	0,04	Banks	-	0,03	0,02	0,07	-
Volatility	-	0,02	0,06	0,01	-	Volatility	-	0,02	0,02	0,07	0,02
Sovereign	-	-	-	-	-	Sovereign	-	0,03	-	-	0,06
Italy											
IN	FX	Index	Banks	Volatility	Sovereign	OUT	FX	Index	Banks	Volatility	Sovereign
FX	0,18	0,01	0,23	-	-	FX	0,16	0,19	-	0,19	-
Index	-	0,09	-	-	0,08	Index	-	0,11	0,05	-	-
Banks	-	0,05	-	0,08	0,01	Banks	0,01	0,12	0,02	-	-
Volatility	0,01	0,23	-	0,02	-	Volatility	-	0,02	0,04	0,05	0,03
Sovereign	-	-	-	-	-	Sovereign	-	-	-	-	-
China											
IN	FX	Index	Banks	Volatility	Sovereign	OUT	FX	Index	Banks	Volatility	Sovereign
FX	0,25	-	-	-	-	FX	-	-	0,19	0,13	-
Index	-	0,03	0,02	-	-	Index	-	-	-	0,04	-
Banks	0,09	0,03	0,08	0,07	0,01	Banks	-	0,29	0,02	0,09	-
Volatility	-	-	-	-	-	Volatility	-	-	-	0,04	-
Sovereign	0,10	0,12	-	0,01	0,16	Sovereign	-	0,11	-	-	0,08
Japan											
IN	FX	Index	Banks	Volatility	Sovereign	OUT	FX	Index	Banks	Volatility	Sovereign
FX	0,07	-	-	0,10	-	FX	0,53	-	-	-	-
Index	-	0,06	0,38	0,05	0,05	Index	0,25	-	-	-	-
Banks	-	0,07	0,03	0,10	0,06	Banks	0,01	0,06	-	-	0,04
Volatility	-	-	0,01	0,01	0,01	Volatility	-	-	-	-	-
Sovereign	-	-	-	-	-	Sovereign	0,11	-	-	-	-
Australia											
IN	FX	Index	Banks	Volatility	Sovereign	OUT	FX	Index	Banks	Volatility	Sovereign
FX	-	-	-	-	-	FX	-	-	-	0,27	0,32
Index	0,12	0,09	0,12	0,01	-	Index	-	-	0,12	-	0,02
Banks	-	0,19	0,06	0,11	-	Banks	-	-	0,06	-	0,02
Volatility	0,06	-	0,02	-	0,05	Volatility	-	-	-	-	-
Sovereign	0,01	0,05	0,05	-	0,06	Sovereign	-	-	-	-	0,18

Looking now at the Stock market indices, we see that the influence of this variable is especially high for the European countries for both information inflows and outflows but also for Japan and Australia for the information inflows. In contrast with the results obtained by [4] which demonstrates the importance of the U.S. equity market as a stress transmitter to other equity markets, when looking at the results of our model, the U.S. equity market does not seem to play an important role in the transmission of information as this channel represents only 2 percent.

With regards the financial institutions, our results confirm the importance of the U.K. and U.S. financial sectors as transmitters of risk. The financial sector of the countries from the Asia-Pacific region seems to be the most vulnerable to information coming from other countries especially the Australian one. Australia is also, with the U.S., the country which influenced the most the others with respect to their sovereign bond market. Germany is also a country for which the bond market has a relatively high importance in terms of incoming information flows, especially from the foreign equity market with both the volatility and stock market indices influencing its sovereign bond market. We observed eventually that, except for Italy, countries transmitting a lot of information with their volatility index, are countries for which the volatility index is the least important parameters for information inflows.

After having considered aggregated results, we now turn to a more detailed description by looking at the main channel of communication for each pair of connected countries. As can be seen from Table 4, the results confirm the major role of the currencies in the transmission of information to North America while the information transmission inside the European Union is mainly driven by the financial and stock markets both in terms of returns and volatilities. The transmissions from the European Union to Asia involve two main channels, a Currency-Currency channel and a Financial market-Stock market channel, highlighting the importance of the Euro and the European financial industry in Asia. The Asian financial markets and currencies, in turn, tends to influence the European countries. As regards the links between North America and Europe, we see that the European markets are very attentive to North American indicators.

**Table 4:** This table reports for each pair of connected countries, the main channel driven the information transmission, with the horizontal axis reporting the countries transmitting the information and the vertical axis reporting the countries receiving the information (with FX for the currencies, FI for the financial indices, Ind for the stock market indices, Vol for the volatility indices and Sov for the sovereign bonds).

Rec/Trans	US	CA	UK	DE	FR	IT	CN	JP	AU
US	0	FX-Sov	0	FX-FX	FX-FX	FX-Vol	FI-FI	FX-FX	FX-Vol
CA	FX-FX	0	FX-FX	FX-Ind	Vol-Vol	FX-FX	0	FX-FX	FX-Sov
UK	0	FX-Sov	0	Ind-Ind	Sov-Sov	FX-Ind	FI-Ind	FX-FX	FX-Vol
DE	FX-Sov	Ind-FX	Sov-Vol	0	Ind-Ind	Vol-Ind	Sov-Ind	0	FX-Sov
FR	Ind-Vol	Ind-Ind	0	Ind-Ind	0	Vol-FI	FI-Vol	Ind-FX	FI-FI
IT	Ind-Ind	Ind-Sov	FI-Vol	Vol-Ind	Vol-Vol	0	FX-FI	FX-FX	0
CN	Sov-Ind	FI-FX	FI-FI	FX-FX	FX-FX	Ind-Ind	0	Sov-FX	Sov-Sov
JP	Ind-FI	0	Ind-FI	Ind-FI	Ind-FI	FX-FX	FX-Vol	0	Ind-FI
AU	0	Ind-Ind	Vol-FX	Ind-FI	Sov-Ind	Ind-FI	FI-Vol	Ind-FX	0

### Commodity markets and cross-country information transfers

We investigate in this section the role of commodity markets in the transmission of information between countries. The past decade has witnessed a strong modification of commodities’ connections to the financial market, as they have been gradually included in many portfolios of financial market players such as hedge funds and day traders for a risk diversification purpose. This financialization of commodities [39, 40] explains the large fluctuation observed in the market during the last global financial crisis. These fluctuations affected many countries leading to a capital outflow for exporting countries when the prices decreased due to the slowdown of emerging market economy; and reducing their competitiveness when the prices increased via currency appreciation. Commodities fluctuations have also impacted financial system as illustrated by the recent drop in crude oil price which jeopardizes many investments of financial institutions in new exploration projects, affecting the stock market. These effects illustrates clearly the influence of commodities in the evolution of the economy of both importing and exporting countries. Commodities could therefore represent an additional channel of communication between importing and exporting countries.

The topics addressed by the literature treating about commodities can be grouped into three major categories, the papers that focus on the interdependencies between commodity markets [41, 42], the ones that examine the spillovers between commodities and financial variables such as exchange rates, interest rates or stock prices [11] and the ones that model the volatility of these commodities [43, 44]. In the second type of approach, exchange rates are treated as the most important macroeconomic variable and could therefore be, as mentioned earlier, the preferred channel of transmission between countries and the commodity markets in our analysis. This section proposes therefore to contribute to this second strand of the literature about commodities.

### Analysis of commodity markets and cross-country information transfers

We rely, in this section, on a similar procedure as the one developed for cross-country interdependencies detection, in order to assess the information flows from the commodity markets to the different countries and from the countries to the commodity markets. We create therefore a commodity system represented by the 8 variables presented earlier, gold, silver, aluminum, copper, crude oil, soybean, wheat and corn. We start by estimating the active channel to be considered in our multichannel causality measure by applying once again a bivariate Granger causality test. We then apply our model and average the probability of activity for each channel for the whole sample and for the different periods mentioned before.

We then start by aggregating the information for all the countries to get a global overview of the inflows and outflows for the commodities. As can be seen from Table 5, the commodities seems to impact mostly the currencies with for the entire period, 52 percent of the total information flow imputable to the channel Commodity-Currency. This clearly confirms the importance of the relationship between these two markets and reinforce the conclusions of the abundant literature treating this subject [11, 45]. The same is true for the stock market indices with 28 percent overall. With regards the distinctive features of each commodity, gold, metals and oil have clearly a much higher influence than food on advanced markets. Gold has the higher impact with 43 percent of the total information flows, influencing mainly the evolution of exchange rates and stock market indices. This high percentage and the impact on stock market indices is mainly due to its role of safe haven asset, especially in time of crisis. As for the other metals, their impact on currencies is mainly due to the importance of theses raw materials for both importing and exporting countries’ economy. They seem to have a more global impact than oil which influence only the stock market indices. Similarly to the results obtained for the information flows between countries, we observed a great stability in time for the channels repartition with only small variations when considering the different periods.

**Table 5:** This table reports for the different periods, the main channels used for the transfer of information from the commodity market to the countries of our data-set with the horizontal axis reporting the variables of the commodity market transmitting the information and the vertical axis reporting the variables of the countries to which the information is transmitted.

Entire Period								
OUT	Gold	Silver	Aluminium	Copper	Oil	Soybean	Wheat	Corn
FX	0,24	-	0,12	0,16	-	0,01	-	-
Index	0,15	-	0,03	-	0,07	0,03	-	-
Banks	0,05	-	-	0,04	-	-	-	-
Volatility	-	0,01	0,02	-	0,03	-	-	0,01
Sovereign	-	-	0,03	-	-	-	-	-
Pre-Crisis Period								
OUT	Gold	Silver	Aluminium	Copper	Oil	Soybean	Wheat	Corn
FX	0,25	-	0,12	0,17	-	0,01	-	-
Index	0,12	-	0,03	-	0,05	0,02	-	-
Banks	0,03	-	-	0,06	-	-	-	-
Volatility	-	0,01	0,05	-	0,05	-	-	0,01
Sovereign	-	-	0,02	-	-	-	-	-
Global Financial Crisis Period								
OUT	Gold	Silver	Aluminium	Copper	Oil	Soybean	Wheat	Corn
FX	0,25	0,01	0,13	0,16	-	0,01	-	-
Index	0,14	-	0,03	-	0,07	0,03	-	-
Banks	0,02	0,01	-	0,03	-	-	-	0,01
Volatility	-	0,01	0,01	-	0,03	-	-	0,01
Sovereign	-	-	0,04	-	-	-	-	-
European Sovereign Debt Crisis Period								
OUT	Gold	Silver	Aluminium	Copper	Oil	Soybean	Wheat	Corn
FX	0,22	-	0,11	0,15	-	0,01	-	-
Index	0,18	-	0,03	-	0,08	0,04	-	-
Banks	0,07	-	-	0,03	-	-	-	0,01
Volatility	-	0,01	-	-	0,01	-	-	-
Sovereign	-	-	0,03	-	-	-	-	-
Post-Crisis Period								
OUT	Gold	Silver	Aluminium	Copper	Oil	Soybean	Wheat	Corn
FX	0,22	-	0,10	0,15	-	0,01	-	-
Index	0,17	-	0,04	-	0,08	0,04	-	-
Banks	0,09	-	-	0,04	-	-	-	-
Volatility	-	0,01	-	-	0,01	-	-	-
Sovereign	-	-	0,03	-	-	-	-	-

Table 6 provides information about the impact of countries fundamentals on the commodity markets. In contrast with the major role of gold for the currencies and the equities, we found no information transmitted from these different markets to gold. Instead, it seems that copper and oil are highly influenced by the selected financial variables. Nevertheless, this does not mean that these variables have no influence on gold but just that this influence is less pronounced than for copper or oil. Overall the commodities are mainly influenced by the exchange rate and by the equity market. As for the evolution in time, we observed the same stability as before.

**Table 6:** This table reports for the different periods, the main channels used for the transfer of information from the countries of our data-set to the commodity market with the horizontal axis reporting the variables of the countries transmitting the information and the vertical axis reporting the variables of the commodity market to which the information is transmitted.

Pre-Crisis					
IN	FX	Index	Banks	Volatility	Sovereign
Gold	-	-	-	-	-
Silver	-	0,02	0,03	-	-
Aluminium	-	0,09	-	-	-
Copper	0,20	0,22	0,05	-	-
Oil	0,20	-	-	-	-
Soybean	-	-	-	-	-
Wheat	-	-	0,04	-	-
Corn	-	0,09	-	0,05	-
Post-Crisis					
IN	FX	Index	Banks	Volatility	Sovereign
Gold	-	-	-	-	-
Silver	-	0,02	0,03	-	-
Aluminium	-	0,10	-	-	-
Copper	0,20	0,22	0,05	-	-
Oil	0,20	-	-	-	-
Soybean	-	-	-	-	-
Wheat	-	-	0,04	-	-
Corn	-	0,09	-	0,05	-
Post-Crisis					
IN	FX	Index	Banks	Volatility	Sovereign
Gold	-	-	-	-	-
Silver	-	0,02	0,03	-	-
Aluminium	-	0,08	-	-	-
Copper	0,20	0,21	0,05	-	-
Oil	0,20	-	-	-	-
Soybean	-	-	-	-	-
Wheat	-	-	0,04	-	-
Corn	-	0,10	-	0,05	-
Post-Crisis					
IN	FX	Index	Banks	Volatility	Sovereign
Gold	-	-	-	-	-
Silver	-	0,02	0,03	-	-
Aluminium	-	0,08	-	-	-
Copper	0,20	0,23	0,05	-	-
Oil	0,20	-	-	-	-
Soybean	-	-	-	-	-
Wheat	-	-	0,05	-	-
Corn	-	0,09	-	0,05	-
Post-Crisis					
IN	FX	Index	Banks	Volatility	Sovereign
Gold	-	-	-	-	-
Silver	-	0,02	0,03	-	-
Aluminium	-	0,10	-	-	-
Copper	0,20	0,23	0,05	-	-
Oil	0,20	-	-	-	-
Soybean	-	-	-	-	-
Wheat	-	-	0,04	-	-
Corn	-	0,09	-	0,05	-

In order to assess the possible use of commodity markets as an additional indirect channel for information transmission between the countries of our data-set, we now consider, in more details, the relationships existing between the selected financial variables and the commodities for each country. To be able to compare properly the strength of these relationships, we create five new data-sets, one for each type of financial variables. These data-sets regroups for each financial variable the time series of every countries. We then estimate using the same methodology as before the links existing between the commodities and each of these data-set, looking at relationships from the commodities to the financial variables and from the financial variables to the commodities.

The resulting matrices are given in Table 7 for the transmission from the selected financial variables to the commodities and in Table 8 for the transmission in the reverse direction. As can be seen for Table 7, apart from the exchange rate, we observe a transmission of information from the countries’ variables to the commodities for North America and Europe, with food commodities mainly impacted by Europe and metal and oil by North America. This is in line with the fact that the U.S. and Canada are large producers of oil and metals and that Europe contributes to the commodity market mainly via food production. When looking now at Table 8, we observe a clear transmission from the commodity markets to the Asian financial variables, with metals, oil and food impacting their fundamentals. This tends to prove that, in addition to the cross-country channels highlighted in section 1, there seems to exist a channel from North America to Asia using metals and oil as transmitter and from Europe to Asia using food supply as transmitter. As for the exchange rate, we see clearly that the commodities influence mainly the USD which may be explained by the fact that most commodities, especially metals, are traded in USD and therefore their prices influence the demand for this currency and in turn its value [11].

**Table 7:** This table reports for the entire period, the main channels through which the financial variables of the selected countries influence commodities.

Currencies							
FX	USD	CAD	GBP	EUR	CNY	JPY	AUD
Gold	-	-	-	-	-	-	-
Silver	-	-	-	-	-	-	-
Aluminium	-	-	0,95	-	-	-	-
Copper	-	0,02	-	0,02	-	-	-
Oil	-	-	-	-	-	-	-
Soybean	-	-	-	-	-	-	-
Wheat	-	-	-	-	-	-	-
Corn	-	-	-	-	-	-	-

Stock market indices									
Equi	U.S.	CA	U.K.	DE	FR	IT	CN	JP	AU
Gold	0,31	-	-	-	-	-	-	-	-
Silver	-	-	-	-	-	-	-	-	-
Aluminium	0,42	-	-	-	-	-	-	-	-
Copper	-	-	-	-	-	-	-	-	-
Oil	0,27	-	-	-	-	-	-	-	-
Soybean	-	-	-	-	-	-	-	-	-
Wheat	-	-	-	-	-	-	-	-	-
Corn	-	-	-	-	-	-	-	-	-
Financial indices									
FI	U.S.	CA	U.K.	DE	FR	IT	CN	JP	AU
Gold	-	-	-	-	-	-	-	-	-
Silver	-	-	-	-	-	-	-	-	-
Aluminium	-	-	-	-	-	-	-	-	-
Copper	-	-	-	-	-	-	-	-	-
Oil	-	-	-	-	-	-	-	-	-
Soybean	-	-	-	-	-	-	-	-	-
Wheat	-	-	-	0,51	-	-	-	-	-
Corn	-	-	0,04	-	-	0,45	-	-	-
Volatility indices									
Vol	U.S.	CA	U.K.	DE	FR	IT	CN	JP	AU
Gold	-	-	-	-	-	-	-	-	-
Silver	0,29	-	-	-	-	-	-	-	-
Aluminium	0,38	-	-	-	-	-	-	-	-
Copper	0,33	-	-	-	-	-	-	-	-
Oil	-	-	-	-	-	-	-	-	-
Soybean	-	-	-	-	-	-	-	-	-
Wheat	-	-	-	-	-	-	-	-	-
Corn	-	-	-	-	-	-	-	-	-
Sovereign bond yield									
Sov	U.S.	CA	U.K.	DE	FR	IT	CN	JP	AU
Gold	-	-	-	-	-	-	-	-	-
Silver	-	-	-	-	-	-	-	-	-
Aluminium	-	-	-	-	-	-	-	-	-
Copper	-	-	-	-	-	-	-	-	-
Oil	-	1,00	-	-	-	-	-	-	-
Soybean	-	-	-	-	-	-	-	-	-
Wheat	-	-	-	-	-	-	-	-	-
Corn	-	-	-	-	-	-	-	-	-

**Table 8:** This table reports for the entire period, the main channels through which the financial variables of the selected countries are influenced by commodities.

Currencies								
FX	Gold	Silver	Aluminium	Copper	Oil	Soybean	Wheat	Corn
USD	0,82	-	-	0,17	-	-	-	-
CAD	-	-	-	-	-	-	-	-
GBP	0,01	-	-	-	-	-	-	-
EUR	-	-	-	-	-	-	-	-
CNY	-	-	-	-	-	-	-	-
JPY	-	-	-	-	-	-	-	-
AUD	-	-	-	-	-	-	-	-
Stock market indices								
Equi	Gold	Silver	Aluminium	Copper	Oil	Soybean	Wheat	Corn
U.S.	-	-	-	-	-	-	-	-
CA	-	-	-	-	-	-	-	-
U.K.	-	-	-	-	-	-	-	-
DE	-	-	-	-	-	-	-	-
FR	-	-	-	-	-	-	-	-
IT	-	-	-	-	-	-	-	-
CN	-	-	-	-	-	-	-	-
JP	-	-	0,11	-	-	-	0,21	-
AU	-	-	0,31	0,05	0,32	-	-	-
Financial indices								
FI	Gold	Silver	Aluminium	Copper	Oil	Soybean	Wheat	Corn
U.S.	-	-	-	-	-	-	-	-
CA	-	-	-	-	-	-	-	-
U.K.	-	-	-	-	-	-	-	-
DE	-	-	-	-	-	-	-	-
FR	-	-	-	-	-	-	-	-
IT	-	-	-	-	-	-	-	-
CN	-	-	0,31	-	-	-	0,65	-
JP	-	-	-	-	-	-	-	-
AU	-	-	-	-	-	-	-	0,03
Volatility indices								
Vol	Gold	Silver	Aluminium	Copper	Oil	Soybean	Wheat	Corn
U.S.	-	-	-	-	-	-	-	-
CA	-	-	-	-	-	-	-	-
U.K.	-	-	-	-	-	-	-	-
DE	-	-	-	-	-	-	-	-
FR	-	-	-	-	-	-	-	-
IT	-	-	-	-	-	-	-	-
CN	-	-	-	-	-	-	-	-
JP	-	0,02	0,03	0,01	0,08	0,11	0,30	-
AU	-	0,30	-	-	-	-	-	0,15
Sovereign bond yield								
Sov	Gold	Silver	Aluminium	Copper	Oil	Soybean	Wheat	Corn
U.S.	-	-	-	-	-	-	-	-
CA	-	-	-	-	-	-	-	-
U.K.	-	-	-	-	-	-	-	-
DE	-	-	-	-	-	-	-	-
FR	-	-	-	-	-	-	-	-
IT	-	-	-	-	-	-	-	-
CN	-	-	-	-	0,44	-	-	-
JP	-	-	-	-	-	-	-	-
AU	0,26	-	0,28	-	0,01	0,02	-	-

As we considered, in our analysis, several different times series in a single measure, co-movements in the set of selected fundamentals may alter the results of our analysis. Therefore, we repeated the analysis presented in the previous sections, by filtering the raw data from common factor using for each country the first principal component of the set of fundamentals characterizing the country. The results are qualitatively the same and available upon request.

## Conclusion

In this paper, we explored the possibility of improving our understanding of the information flows inside the economy taking into account the greater interdependence of markets due to the financial globalization. We proposed a new approach derived from the Markov Switching model to infer the information transmissions between different multivariate systems. Beyond the global measure proposed by [21] and the Granger causality test developed by [23], our approach provides a detailed view of the channels’ dynamics describing the information transmission process between two complex systems, providing information on the channels’ activity at each time step.

We investigated using our new methodology the causal relationships existing between 9 countries representing the world major economies. Instead of looking at simple pairwise relationships, our new methodology enables us to look at the same time at several channels of transmission by considering for each country 5 different financial variables that have been selected to represent the state of each country.

The aggregated results demonstrated the importance of currencies and equities as both transmitters and receivers of information in contrast with the common view that since the Global Financial Crisis, the main vectors of information were the financial institutions. Nevertheless, the U.S. and U.K. financial sector have proven to be relatively important sources of information. As for the currency channel, the Euro and the Japanese Yen seem to be the main spreader of information influencing mainly the U.S. dollar and the Canadian dollar. Eventually, the channels involving the stock market indices were especially important inside the European Union.

The second part of our empirical investigation consisted in examining the relationships existing between countries and the commodity markets and look at the possibility that the commodity markets could represent an additional indirect channel of information transmission between countries. Our results confirmed the major relationships existing between commodities and exchange rates but also between commodities and equities. These close ties concern primarily metals and oil. Eventually we have determined the existence of a link between North America and Asia through oil and metals and from Europe to Asia through agricultural and food commodities.

## Supporting information

S1 Dataset Raw data regrouping the economic and financial variables used in the results and discussion section of the paper. (ZIP)
